# Transcriptome and proteome analysis of *Salmonella enterica* serovar Typhimurium systemic infection of wild type and immune-deficient mice

**DOI:** 10.1371/journal.pone.0181365

**Published:** 2017-08-10

**Authors:** Olusegun Oshota, Max Conway, Maria Fookes, Fernanda Schreiber, Roy R. Chaudhuri, Lu Yu, Fiona J. E. Morgan, Simon Clare, Jyoti Choudhary, Nicholas R. Thomson, Pietro Lio, Duncan J. Maskell, Pietro Mastroeni, Andrew J. Grant

**Affiliations:** 1 Department of Veterinary Medicine, University of Cambridge, Cambridge, United Kingdom; 2 Computer Laboratory, University of Cambridge, JJ Thomson Avenue, Cambridge, United Kingdom; 3 Wellcome Trust Sanger Institute, Wellcome Trust Genome Campus, Hinxton, Cambridge, United Kingdom; 4 The London School of Hygiene and Tropical Medicine, London, United Kingdom; Universitat Osnabruck, GERMANY

## Abstract

*Salmonella enterica* are a threat to public health. Current vaccines are not fully effective. The ability to grow in infected tissues within phagocytes is required for *S*. *enterica* virulence in systemic disease. As the infection progresses the bacteria are exposed to a complex host immune response. Consequently, in order to continue growing in the tissues, *S*. *enterica* requires the coordinated regulation of fitness genes. Bacterial gene regulation has so far been investigated largely using exposure to artificial environmental conditions or to *in vitro* cultured cells, and little information is available on how *S*. *enterica* adapts *in vivo* to sustain cell division and survival. We have studied the transcriptome, proteome and metabolic flux of *Salmonella*, and the transcriptome of the host during infection of wild type C57BL/6 and immune-deficient *gp91*^-/-^*phox* mice. Our analyses advance the understanding of how *S*. *enterica* and the host behaves during infection to a more sophisticated level than has previously been reported.

## Introduction

*Salmonella enterica* is a facultative intracellular pathogen capable of causing a spectrum of diseases in humans and animals. *S*. *enterica* serovar Typhi causes ~22 million cases of typhoid fever and over 200,000 deaths annually; with an additional estimated ~5.5 million annual cases of paratyphoid fever in humans [1]. Other non-typhoidal *Salmonella* serotypes (NTS) cause gastroenteritis in humans and animals and can spread from animals to humans *via* contaminated food. NTS are a common cause of bacteraemia and sepsis in immune-compromised individuals and in children, especially in developing countries, where they constitute a major cause of death; no licensed vaccines against NTS are available [2–5]. The emergence of multi-drug resistant *Salmonella* strains and the lack or insufficient efficacy of the currently available *Salmonella* vaccines highlight the urgent need for improved prevention strategies to combat salmonellosis in humans and animals. Understanding how the pathogen grows and adapts during the infection process could offer insights into novel interventions. In this regard, the regulation of *S*. *enterica* gene expression in defined media and cultured cells is being studied. For example, Srikumar *et al*. [6] compared the intra-macrophage transcriptome of *S*. Typhimurium after 8 hours of infection of murine macrophages to early stationary phase *in vitro* grown bacteria. However, *in vitro* experiments offer only a simplistic model of the disease process. Currently, it is not possible to mimic *in vitro* the many, possibly unknown, inflammatory events that occur during infection. The mouse is a tractable and widely used *in vivo* model that has contributed to our understanding of innate and acquired immunity to salmonellosis and supported vaccine development [7, 8].

In the present study, we looked at the system as a whole, assessing the transcriptome of both host and pathogen, as well as the bacterial metabolic flux and proteome during infection. The datasets generated increase our understanding of *S*. Typhimurium and the host during infection, and the approach that we have taken is applicable to other host-pathogen combinations.

## Results and discussion

### Determining the transcriptome of *S*. Typhimurium *in vivo*

**Fig 1:**
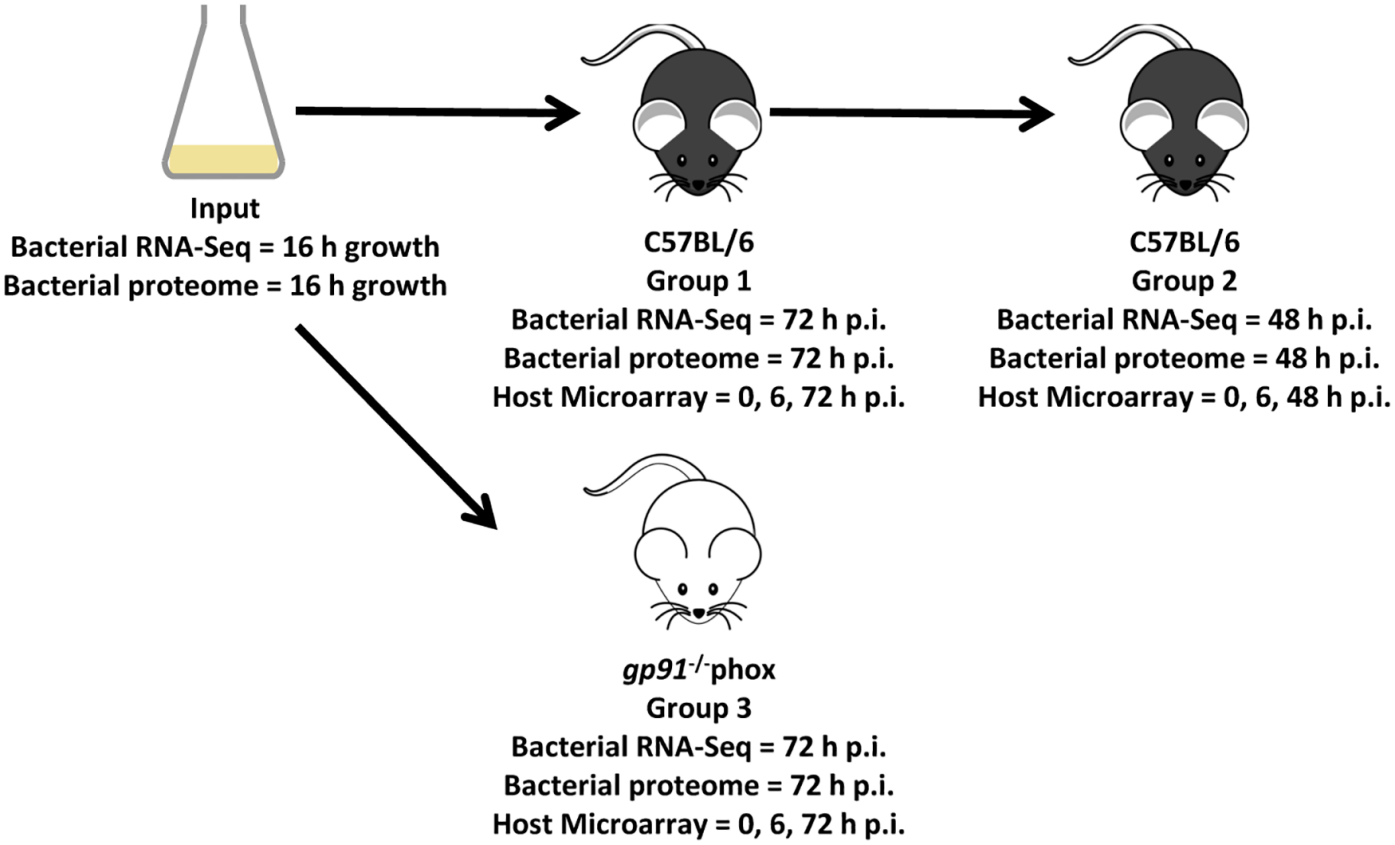
Figure to show the different experimental conditions. The figure shows the three different *in vivo* Groups and *in vitro* grown Input, as well as indicating the time points for the transcriptome (RNA-Seq) and proteome analysis for the bacteria, and transcriptome (microarray) analysis of the host.

In order to characterise the transcriptome of *S*. Typhimurium SL1344 in different host-pathogen combinations, three groups of mice were infected intravenously (i.v.) (Fig 1 and Table 1). The different experimental conditions are hereafter referred to as Group 1, Group 2 and Group 3. Group 1 represents wild type C57BL/6 mice infected with virulent *S*. Typhimurium SL1344 grown *in vitro*; a model commonly used to study systemic *S*. Typhimurium infection. Group 2 represents wild type C57BL/6 mice infected with virulent *S*. Typhimurium SL1344 grown *in vivo* for 72 h in the Group 1 C57BL/6 mice; since we have recently shown that *in vivo* passaged bacteria have an increased net growth rate and an altered death rate in a recipient mouse [9, 10]. Group 3 represents immune-deficient *gp91*^-/-^*phox* mice infected with virulent *S*. Typhimurium SL1344 grown *in vitro*; since we were interested in discovering how the bacteria and the host respond when the immune system is impaired. *gp91* encodes one of the subunits of the NADPH oxidase, an enzyme essential for reactive oxygen species (ROS) production by phagocytes. ROS deficiency leads to reduced initial killing of the bacteria and accelerated growth in the first few days of infection [11, 12].

**Table 1:** Experimental conditions, sample names and definitions.

Condition	Description	Inoculum (Log_10_ CFU)	Count in Spleen (mean Log_10_ CFU +/- Std Dev)	Time (h.p.i.)	Total mapped reads
Input	*S*. Typhimurium SL1344 grown in LB broth, standing, for 16h and used as the inoculum	N/A	N/A	N/A	44475893
Group 1	C57BL/6 wild type mice infected by i.v. injection with *S*. Typhimurium SL1344 grown in LB, standing for 16	4.00	7.19 (0.22)	72	331948
Group 2	C57BL/6 wild type mice infected by i.v. injection with *S*. Typhimurium SL1344 grown in vivo in C57BL/6 wild type mice for 72 h and recovered from the spleens (Group 1)	4.63	7.68 (0.28)	48	1408080
Group 3	*gp91*^*-/-*^*phox* mice infected by i.v. injection with *S*. Typhimurium SL1344 grown in LB, standing for 16 h	2.2	8.34 (0.12)	48	1993042

At appropriate times during the infection, when the bacterial load in the organs was approximately equivalent for each group (Table 1), the mice were killed and total bacterial RNA was isolated from the spleens, as well as from the input bacteria. The 16S and 23S rRNA species were depleted prior to sequencing using selective capture and magnetic separation. The resulting RNA was reverse transcribed into cDNA that was then processed into a library of molecules that could be sequenced on an Illumina HiSeq. The sequence reads were mapped to the genome sequence of SL1344 (GenBank ID: FQ312003). The total number of reads obtained and mapped for each sample is detailed in Table 1. Full details of the number of reads mapping to each gene and intergenic region are given in S1 Table.

### Comparing the *in vivo* transcriptomes of *S*. Typhimurium with the *in vitro* input

**Fig 2:**
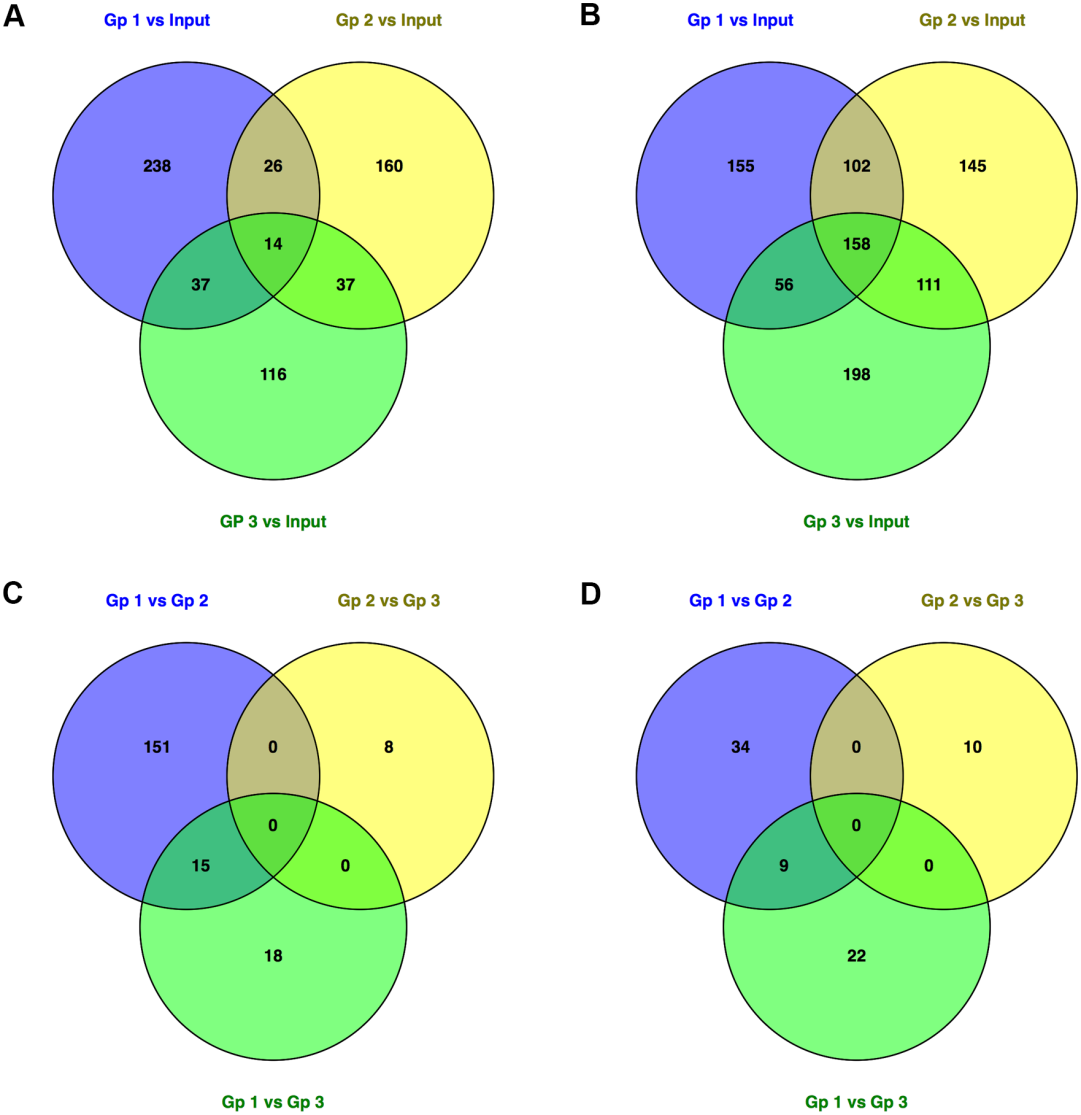
Visualization of the DE bacterial genes. Venn diagrams show (A) Up-regulated DE genes for each Group *vs* the *in vitro* Input. (B) Down-regulated DE genes for each Group *vs* the *in vitro* Input. (C) Up-regulated DE genes for between-Group comparisons. (D) Down-regulated DE genes for between-Group comparisons.

The differentially expressed (DE) genes in *S*. Typhimurium SL1344 from the *in vitro* grown Input and the different *in vivo* Groups, including the pathways that were statistically overrepresented among the DE genes are given in S2 Table. Full lists of the DE genes in *S*. Typhimurium SL1344 from the different experimental combinations can be found in: S3 Table (Group 1 *vs in vitro* Input); S4 Table (Group 2 *vs in vitro* Input); S5 Table (Group 3 *vs in vitro* Input). A summary of the DE genes in *S*. Typhimurium for the Group *vs in vitro* Input comparisons can be seen in S6 Table and Fig 2. Even though the bacterial input for Group 2 was bacteria grown *in vivo*, we have included in our analyses Group 2 *vs in vitro* Input, since we were interested in how the behavior of the bacteria differed once they had been passaged through an animal.

**Fig 3:**
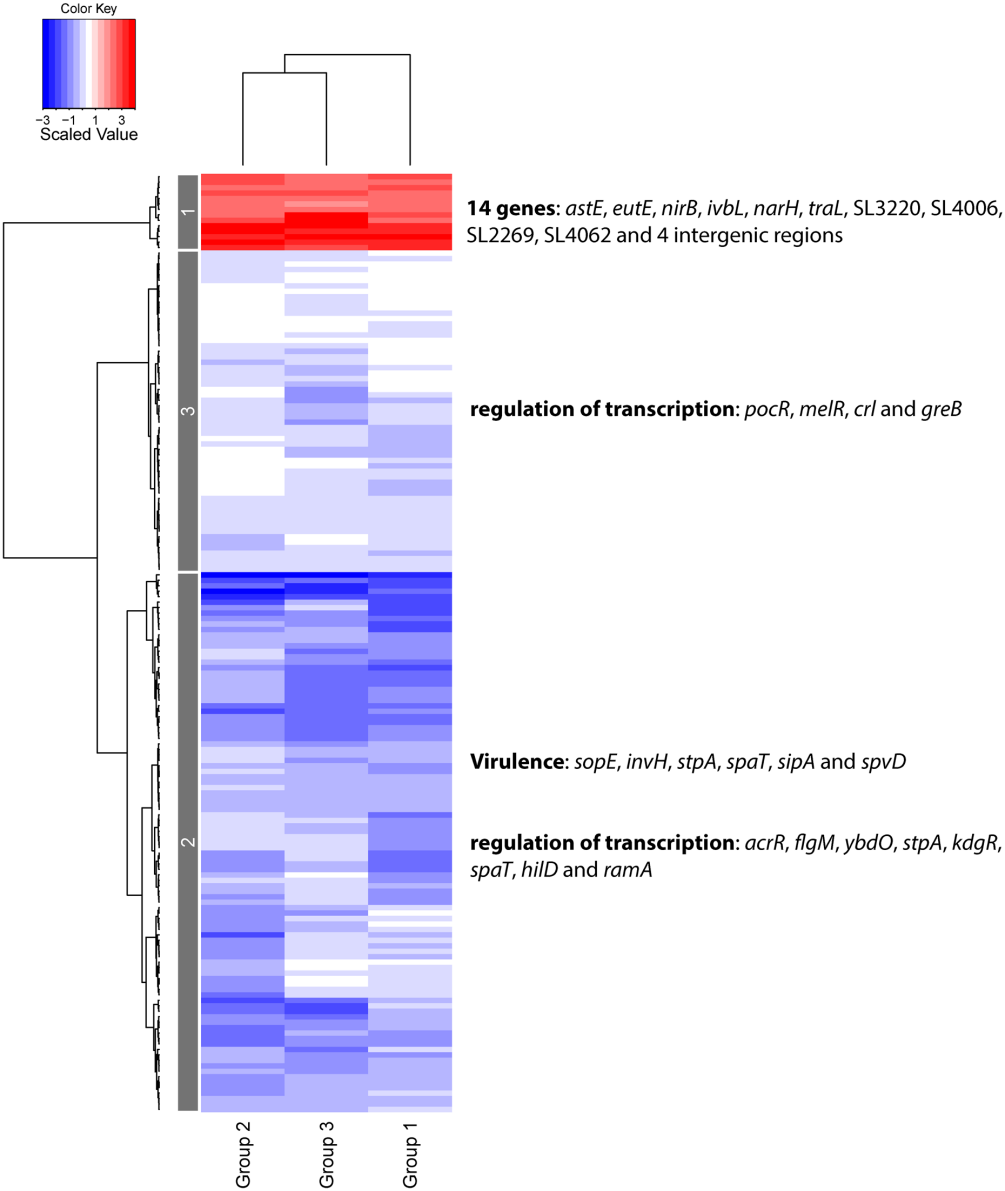
Heatmap of the DE genes shared by *S*. Typhimurium from Groups 1, 2 and 3, relative to the *in vitro* grown Input. The dendrograms are based on Ward’s method using scaling between sample profiles. Red and blue colours represent up- and down-regulation, respectively. Genes are visualized in rows, and Groups are in the columns. Patterns of up- and down-regulated genes are depicted in 3 gene clusters, labelled 1–3. Clusters are annotated with important genes and biological processes.

There were a comparable number of DE genes in each of the three different ‘Group *vs in vitro* Input’ experimental conditions (786 DE genes in Group 1 *vs in vitro* input; 753 DE genes in Group 2 *vs in vitro* input; and 729 DE genes in Group 3 *vs in vitro* input, S2 Table). Our transcriptomic data revealed that 172 DE genes (14 up-regulated and 158 down-regulated) were shared between *S*. Typhimurium in Groups 1, 2 and 3, in comparison to the *in vitro* Input. In order to tease out the important differences between the *in vivo* and *in vitro* conditions in our study, we grouped these shared genes into clusters and the results were visualized as a heatmap (Fig 3).

*S*. Typhimurium recovered from the mice (Groups 1, 2 and 3) had similar gene expression profiles, relative to the *in vitro* grown Input, as indicated by the similar patterns of up-regulated (red) and down-regulated (blue) genes in terms of colour intensities that show the expression of individual genes (Fig 3). Fig 3 also shows that DE genes of *S*. Typhimurium from Group 2 and Group 3 cluster together, indicating that they are closer to each other in terms of their levels of gene expression than to DE genes of *S*. Typhimurium from Group 1. The gene profiles of the three experimental conditions separate into 3 clusters, each with similarly expressed genes (clusters 1–3, Fig 3), two of which represent the down-regulated genes (clusters 2 and 3) and one cluster encompass the up-regulated genes (cluster 1).

Cluster 1 (Fig 3) comprises 14 shared up-regulated genes and intergenic regions in *S*. Typhimurium: *astE* (encodes succinylglutamate desuccinylase; transforms N(2)-succinylglutamate into succinate and glutamate); *eutE* (encodes aldehyde oxidoreductase; may act as an acetaldehyde dehydrogenase that converts acetaldehyde into acetyl-CoA); *ivbL* (encodes IlvB operon leader peptide); *narH* (encodes nitrate reductase 1 beta subunit); *nirB* (encodes nitrite reductase large subunit); *traL* (encodes conjugal transfer pilus assembly protein); SL3220, SL4006, SL2269, SL4062, and four intergenic regions (including one on the plasmid, pSLT1344).

Cluster 2 (Fig 3) includes the *S*. Typhimurium most down-regulated genes in the *in vivo* experimental Groups, compared to the *in vitro* Input. These include *Salmonella* Pathogenicity Island 1 (SPI-1) virulence genes (*sopE*, *invH*, *stpA*, *spaT*, *sipA* and *spvD*), regulation of transcription (*acrR*, *flgM*, *ybdO*, *stpA*, *kdgR*, *spaT*, *hilD* and *ramA*) and flagella-associated processes (*fliC*, *flaG*, *fliB* and *flgM*). The data indicated that SPI-1 genes (required for entry into epithelial cells) were down-regulated in each of the *in vivo* experimental Groups compared to the *in vitro* grown Input. The down-regulation of RamA (encoded by *ramA*; which controls multidrug resistance) as indicated by our data is in line with the previous findings showing that a high expression of this gene generally leads to decreased expression of SPI-2 genes [13], the products of which are required for the systemic phase of the infection [14]. Down-regulation of flagella genes in the *in vivo* data is line with a previous study [15].

Among the cluster 3 genes (Fig 3), are down-regulated genes encoding transcriptional regulators (Crl, GreB MelR, PocR). This suggests a down-regulation of propanediol metabolism, as two operons involved in the regulation of that pathway (*cob* and *pdu*) are regulated by PocR [16].

### Individual comparisons of the transcriptomes of *S*. Typhimurium recovered from mice, for the different groups, with *S*. Typhimurium grown *in vitro*

The relative gene expression of *S*. Typhimurium SL1344 recovered from C57BL/6 mice (Group 1) compared to *in vitro*-grown *S*. Typhimurium SL1344 (Input) indicates the induction of: two-component regulators; genes involved in peptidoglycan biosynthesis; *hmpA*–encoding a soluble flavohemoglobin that counteracts nitrosative stress *in S*. *enterica* and involved in NO detoxification; *entD* (Enterobactin/siderophore biosynthesis) and *sapF* (resistance to antimicrobial peptide) (S3 Table). For the same comparison, there was decreased expression of genes involved in protein biosynthesis; heat shock stress response; oxidative stress response; the synthesis of enzymes involved in aerobic respiration, the TCA cycle, oxidative phosphorylation, electron transport, pyruvate metabolism; and SPI-1 genes (*i*.*e*. implying a lower level of SPI-1 during systemic infection, in-line with previous studies) (S3 Table).

Compared to *S*. Typhimurium grown *in vitro*, *S*. Typhimurium recovered from C57BL/6 mice (Group 2) had increased expression of SPI-2 genes; genes encoding sugar transporters; genes encoding proteins involved in oxidative phosphorylation; genes whose protein products are required for biosynthesis of amino acids; and genes encoding proteins involved in protein biosynthesis (S4 Table). For the same comparison, there was decreased expression of genes encoding: transcriptional regulators; SPI-1; flagella assembly and chemotaxis (*i*.*e*. implying a lower level of flagella expression during systemic infection, in-line with previous studies) [15] (S4 Table).

*S*. Typhimurium from *gp91*^*-/-*^*phox* mice (Group 3) compared to *in vitro*-grown *S*. Typhimurium (Input), showed an increase in expression in genes involved in anaerobic respiration and utilisation of nitrite. The induction of *aceK* suggests that the anaerobic glyoxylate cycle was active. A number of sugar transporter genes were induced and there was increased expression of genes involved in siderophore-mediated iron acquisition and iron-uptake systems (S5 Table). As expected, SPI-1 genes and genes involved in flagella assembly were lower in *S*. Typhimurium from *gp91*^-/-^*phox* mice compared to *in vitro*-grown bacteria (S5 Table).

In line with Srikumar *et al*. [6], who compared the intra-macrophage transcriptome of *S*. Typhimurium after 8 hours of infection of murine macrophages to early stationary phase *in vitro* grown bacteria, our differential gene expression of *in vivo vs in vitro* grown bacteria showed down-regulation of SPI-1 (Groups 1, 2 and 3), flagella assembly (Group 3), and chemotaxis (Groups 2 and 3), and up-regulation of SPI-2 genes (Group 2), and genes involved in amino acid metabolism (Group 2), nitric oxide detoxification (Group 1), and iron uptake (Group 1 and Group 3).

### Pathways that differentiate *Salmonella* between the three experimental groups

Apart from investigating the common transcriptional profiles of *Salmonella* that distinguished the three *in vivo* Groups from the *in vitro* grown (Input), we also determined the unique pathways that differentiate *Salmonella* in the *in vivo* experimental groups from each other, relative to the *in vitro* Input. The results (Fig 2A and 2B, S6 and S7 Tables) suggest that certain pathogenesis-related ABC transporters, namely peptide transport proteins (encoded by *dppC*, *dppF*, *sapF* and *yliC*), cell division protein (encoded by *ftsX*), putrescine ABC transporter membrane protein (encoded by *potH*), phosphate binding protein (encoded by *pstC*) and glycerol-3-phosphate transporter membrane protein (encoded by *ugpE*) were important for *Salmonella* infection in Group 1.

Peptide transport system ATP-binding protein SapF (encoded by *sapF*), a member of the *sapABCDF* operon reported to play a role in the resistance to antimicrobial peptides [17], protein translation (*infB*, *rplB*, *rplD*, *rplF*, *rplO*, *rplP*, *rplV*, *rplW*, *rpmC*, *rpsC*, *rpsS*), energy requirement (*atpA*, *atpD*, *atpF*, and *atpH*) and virulence (*pagC*, *phoP*, *pipB*, *spiC*, *spvA*, *spvB*, *spvC*, *spvR*, *sseC*) were important for *Salmonella* in Group 2 (Fig 2A and 2B, S6 and S7 Tables).

*Salmonella* recovered from Group 3 showed an over-representation of pathways, including siderophore-mediated iron acquisition (*entB*, *entC*, *entE*, *entF*), lipid catabolic process (*fadA*, *fadJ*), and nitrate reductase activity (*napA*, narI, *narJ*, *narY*) (Fig 2A and 2B, S6 and S7 Tables). In *Salmonella*, *entB*, *entC*, *entE*, *entF*, are involved in the synthesis of enterobactin, [18], required for iron acquisition and persistence of infection in mice [19]. Increased expression of the involved in nitrate reductase activity in *Salmonella* in Group 3 may indicate requirement for energy production *via* anaerobic respiration to enhance its intracellular survival [20]. Since the mice in the Group 3 experimental condition are unable to express NADPH oxidase, it is not clear whether increased expression of nitrate reductase plays any role in the protection of *Salmonella* against other forms of stress.

### Comparing bacterial DE genes between experimental groups

A summary of the DE genes in *S*. Typhimurium for the ‘*in vivo* Group vs *in vivo* Group’ comparisons can be seen in S2 and S6 Tables and Fig 2. Full lists of the DE genes in *S*. Typhimurium SL1344 from the different experimental combinations can be found in: S8 Table (Group 1 *vs* Group 2); S9 Table (Group 1 *vs* Group 3); and S10 Table (Group 2 *vs* Group 3). There were far fewer DE genes in the ‘*in vivo vs in vivo’* comparisons compared to the ‘*in vivo vs in vitro* Input’ comparisons (Fig 2 and S2 Table). There were differences in the number of DE genes in each of the three different ‘*in vivo vs in vivo’* experimental conditions (210 DE genes in Group 1 *vs* Group 2; 64 DE genes in Group 1 *vs* Group 3; and 18 DE genes in Group 2 *vs* Group 3; S2 Table).

Pathway analysis revealed that protein translation was significant in the dataset of DE genes down-regulated as indicated by the statistically enriched “Ribosome” pathway (S2 Table); this correlates with the increased net growth rate of the bacteria in Group 2 compared to Group 1.

The number of unique or shared DE genes is given in Fig 2C and 2D and S7 Table, and details of these genes are given in S6 Table. There were no common up- or down-regulated DE genes shared between the groups. Pairwise comparisons between the groups indicated that ‘Group 1 *vs* Group 2’ *vs* ‘Group 1 *vs* Group 3’ was the only comparison where there were shared DE genes, 24 in total; 15 up-regulated and 9 down-regulated (Fig 2C and 2D). The 15 up-regulated DE genes included: *citA* (citrate-protein symporter; involved in the uptake of citrate across the boundary membrane with the concomitant transport of proteins into the cell); *cls* (cardiolipin synthase A; catalyses the reversible phosphatidyl group transfer from one phosphatidylglycerol molecule to form cardiolipin and glycerol); *kbl* (2-amino-3-ketobutyrate coenzyme A ligase; catalyses the cleavage of 2-amino-3-ketobutyrate to glycine and acetyl coA); *sapF* (peptide transport, involved in a peptide transport system that plays a role in the resistance to antimicrobial peptides); *ybiH* (DNA binding transcriptional regulator); as well as eight genes encoding proteins with unknown function, and two intergenic regions. The down-regulated DE genes included: *priB* (primosomal replication protein N; binds single-stranded DNA at the primosome assembly site); *pyrG* (catalyses the ATP-dependent amination of UTP to CTP with either L-glutamine or ammonia as the source of nirtrogen); *udg* (UDP-glucose/GDP-mannose dehydrogenase); as well as 3 genes encoding ribosomal proteins, which is consistent with the decreased net growth rate of Group 1 bacteria compared to Group 2 and Group 3 bacteria—*rplM* (50S ribosomal protein L13; important during the early stages of 50S assembly); *rplU* (50S ribosomal protein L21) *rpsU* (30S ribosomal protein S21); a gene encoding a protein with unknown function and two intergenic regions.

Although the pathway analyses did not identify many pathways as being statistically significantly overrepresented in the different comparisons, there were several individual relevant genes that were significantly up- or down-regulated for each individual grouping, and these are detailed in the following sections.

### Comparing the transcriptome of *S*. Typhimurium from C57BL/6 mice (Group 1) with *S*. Typhimurium from C57BL/6 mice (Group 2)

The results of our gene expression analysis of *S*. Typhimurium show that 43 and 167 genes were up- and down-regulated, respectively, for Group 2 relative to Group 1. The up-regulated genes indicate an induction of genes encoding components of the ribosome and required for biosynthesis of proteins (S8 Table). This correlates with the increased net growth rate of *S*. Typhimurium that had already been passaged through ‘donor’ mice before being introduced into naive mice (Table 1; [9, 10]). Adaptation to stress was indicated in the *S*. Typhimurium from Group 2 due to induction of stress response genes (*grxC*, encoding glutaredoxin 3; *cspC*, encoding cold shock-like protein CspC; *osmY* encoding osmoprotectant import permease protein) and *rfaQ* encoding the lipopolysaccharide (LPS) core biosynthesis protein RfaQ (S8 Table).

The genes more highly expressed in the *S*. Typhimurium from Group 1 compared to *S*. Typhimurium from Group 2 play roles in virulence, starvation stress, utilization of alternative sources of energy, antimicrobial stress, and two-component systems involved in *Salmonella* virulence (S8 Table). *S*. Typhimurium from Group 1 also up-regulated genes encoding transcriptional regulators such as stationary phase inducible protein CsiE (*csiE*), KDP operon transcriptional regulatory protein (*kdpE*), hypothetical tetR-family transcriptional regulator (*ybiH*), pts system fructose-specific IIA/FPR component (*fruB*), ADA regulatory protein (*ada*), transcriptional activator CadC (*cadC*), putative AraC family regulatory protein (*adiY*), transcriptional regulatory protein (*ecnR*) (S8 Table). There was an up-regulation of genes encoding various transporters for extraction of nutrients, including ABC transporters, putative solute-binding proteins including putative PTS systems, and a heavy metal transporter (S8 Table). In addition, there was induction of the anaerobic C4-dicarboxylate transporter (*dcuA*) for extraction of alternative sources of carbon-energy, and genes for ethanolamine utilisation (*eutJ* and *eutN*) (S8 Table).

### Comparing the transcriptome of *S*. Typhimurium from C57BL/6 mice (Group 1) with *S*. Typhimurium from *gp91*^*-/-*^*phox* mice (Group 3)

Differential gene expression analysis indicated 31 up-regulated and 33 down-regulated genes in the comparison of *S*. Typhimurium recovered from the *gp91*^-/-^*phox* mice (Group 3) to *S*. Typhimurium from the C57BL/6 mice (Group 1). Compared to Group 1, the Group 3 *Salmonella* showed an increased the expression of several stress response regulators, and genes involved in protein biosynthesis (*rpsU*, *rplU*, *rplM*, *rpsK*, *rpsJ*, *rpsG*) (S9 Table), which is consistent with the increased net growth rate of *Salmonella* in *gp91*^-/-^*phox* mice compared to C57BL/6 mice (Table 1; [14]). The data suggested that *Salmonella* in the *gp91*^-/-^*phox* mice (Group 3) employed the regulatory homeostatic controls characterised by the induction of genes encoding RpoS and SigmaE, and their negative regulators, LrhA and RseA, respectively (S9 Table). The usage of alternative sources of energy being utilised by *Salmonella* in the *gp91*^-/-^*phox* mice (Group 3) was suggested by the induction of *fdoI* (encoding formate dehydrogenase-O gamma subunit) and the TCA cycle enzymes (S9 Table).

In contrast, *S*. Typhimurium from Group 1 up-regulated genes including those encoding the transcriptional regulatory protein HydG, and the hypothetical *tetR*-family transcriptional regulator YbiH (S9 Table). In addition, genes encoding two enzymes involved in carbohydrate metabolism, 6-phosphofructokinase isozyme (*pfkB*) and putative sugar kinase (*yihV*) were also up-regulated (S9 Table).

### Comparing the transcriptome of *S*. Typhimurium from C57BL/6 mice (Group 2) with *S*. Typhimurium from *gp91*^*-/-*^*phox* mice (Group 3)

Relative to *S*. Typhimurium from Group 2, *S*. Typhimurium from Group 3 had increased expression of 10 genes, among which are those involved in the PTS system (fructose-specific IIA/FPR component encoded by *fruB*), aerobic/anaerobic respiration (probable nitrate reductase encoded by *napA*), iron extraction (putative glycosyltransferase, encoded by *iroB*; and putative ABC transporter protein, encoded by *iroC*), and cell wall modification (UDP-N-acetylmuramoylalanine-D-glutamate ligase encoded by *murD*) (S10 Table).

Gene expression in *S*. Typhimurium from Group 2 relative to *S*. Typhimurium from Group 3, showed induction of 8 genes, including the stress response regulator gene, *ahpC* encoding alkyl hydroperoxide reductase c22 protein, and *ybbN* encoding thioredoxin-like protein (S10 Table), probably to counteract oxidative stress encountered in the host’s cellular environment as a result of production of reactive oxygen species (ROS) not present in the *gp91*^-/-^*phox* mice.

### Metabolic flux based analysis

In order to verify the results of our DE analysis, we used a gene expression constrained flux balance analysis technique similar to those described by Angione *et al*. in [21]. This integrates gene expression with a metabolic network model [22], *via* gene-protein-reaction mappings. The advantage of this over pure DE analysis is that it provides a filtering step. For example, if a linear pathway is largely downregulated, but has one upregulated reaction in it, that reaction may well be an outlier, since although it has the capacity for higher flux, it will in fact be limited by the reactions on each side. Conversely, a single downregulated reaction in an otherwise untouched linear pathway implies that the other connected reactions will be slowed too.

**Fig 4:**
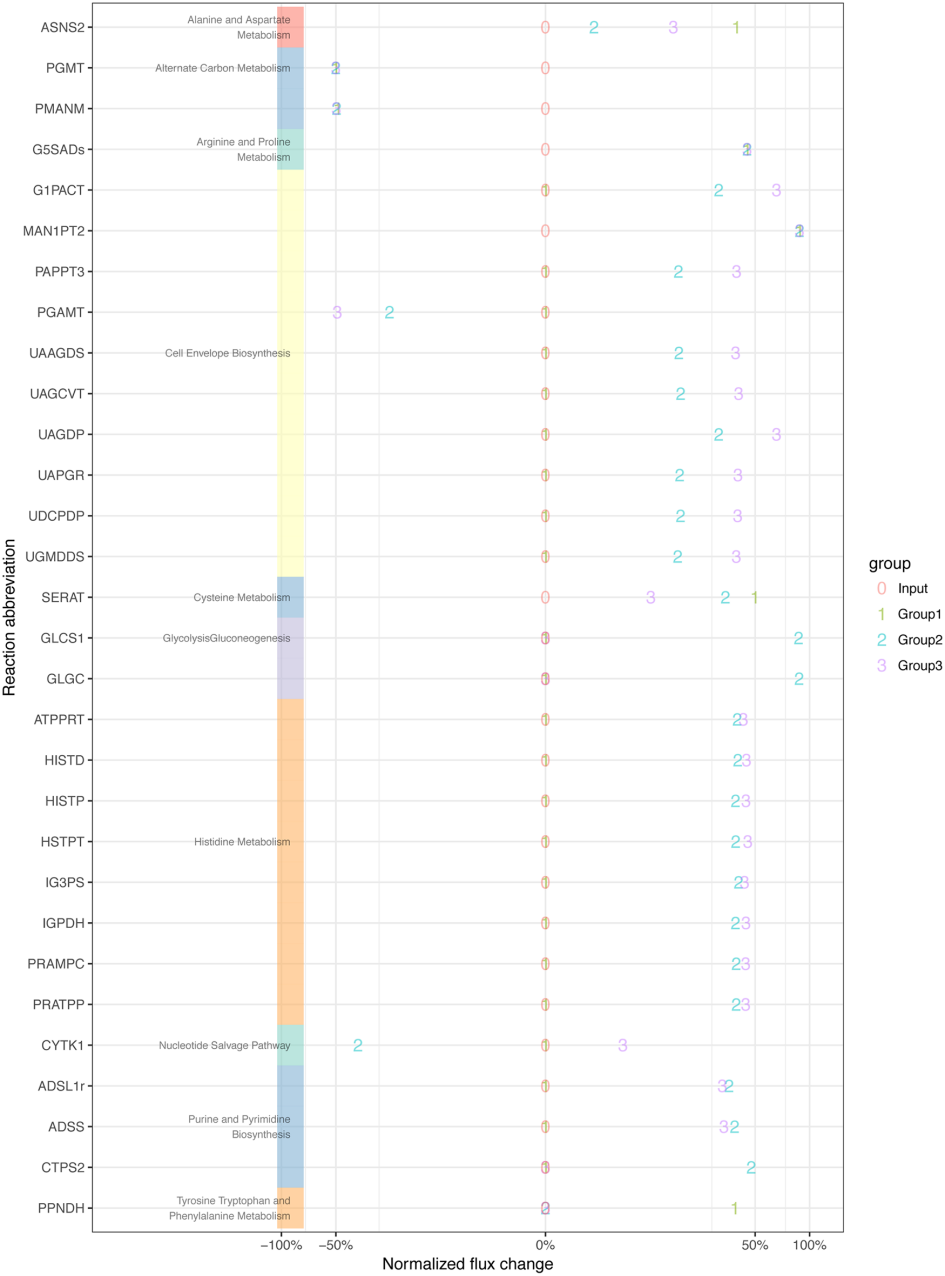
Normalized rates for a number of reactions, selected for the high inter-group variance in their rates. Reaction abbreviations are used in place of reaction names, but a full list can be found in S11 Table. Note that the x-axis scale has been subjected to cubic root scaling to highlight smaller changes. Normalization is against the *in vitro* group (termed Input), hence this value is always at 100%.

**Fig 5:**
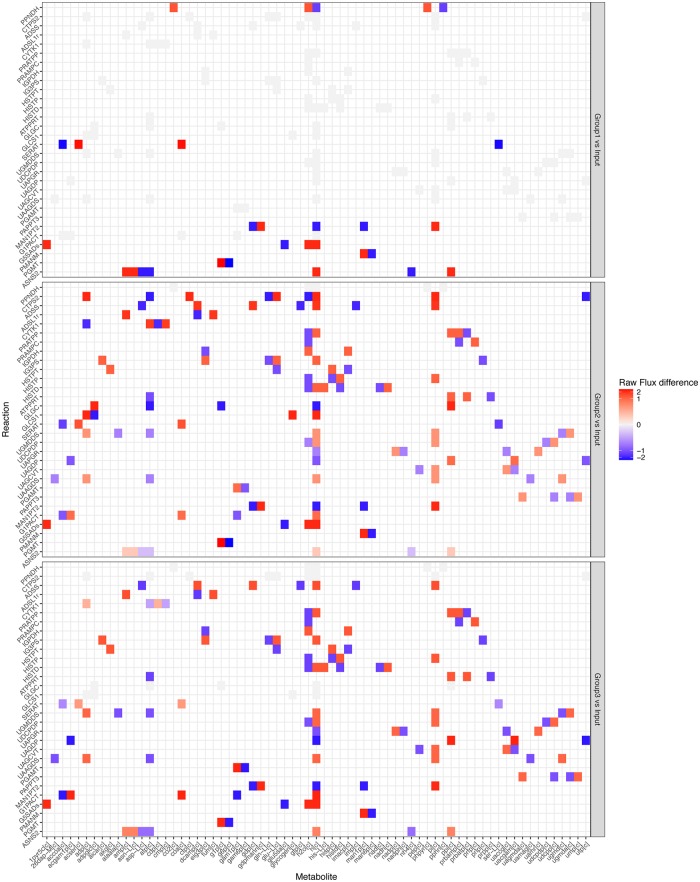
Heatmaps showing the detail of fluxes of each reaction through each metabolite, compared to the *in vitro* case. The flux differences are typically higher than the fluxes in Fig 4, because they are not normalized between groups and are multiplied by the reaction stoichiometry.

**Fig 6:**
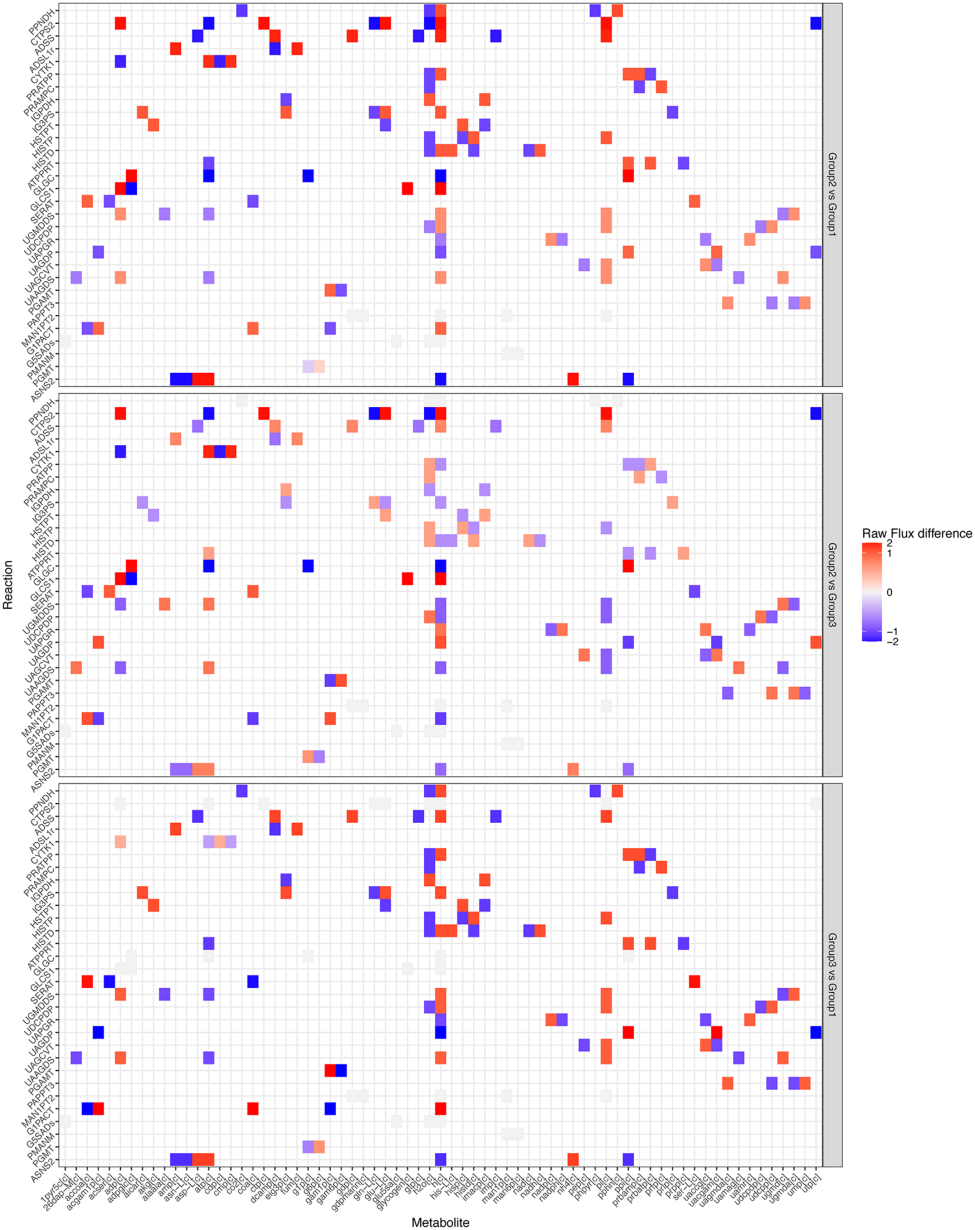
Heatmaps showing a comparison of reactions rates and how the rates interact *via* their associated metabolites. The differences are typically higher than the fluxes in Fig 4, because they are not normalised between Groups and are multiplied by the reaction stoichiometry.

From the 2,500 reactions in the metabolic model, we selected the 30 most interesting, *i*.*e*. those that displayed the largest effect sizes, and had low sensitivity to parameter changes. Fig 4 shows the relative rates of these reactions, while Figs 5 and 6 highlight another useful advantage of this technique, by looking at the metabolites that tie the reactions together, we can see a hint at causality.

In Fig 4, the most obvious pattern is that the Histidine Metabolism, Cell Envelope Biosynthesis pathways are upregulated in Group 2 and Group 3. We might hypothesize that these pathways are upregulated for some combination of repairing immune damage. This pattern is also shown to a lesser extent in Tyrosine Tryptophan and Phenylalanine Metabolism, though in the reaction CTP synthase glutamine (CTPS2), Group 3 is not upregulated. Other reactions and subsystems display a wider variety of behaviours, but we still see the clear and expected pattern that Groups 2 and 3 display more similarity to each other than to Group 1 (Fig 4).

Next we compared metabolic flux differences between individual Groups and the *in vitro* Input (Fig 5). We found that Group 1 displays more polarized responses than Groups 2 and 3, which is due to the fact that it has more in common with the control. In Groups 1 and 2, we can see strong changes around phosphate and mannose metabolism (Fig 5). This is consistent with changes to cell growth and repair pathways (Fig 5). In Group 3, we see changes associated with the enzyme Phospho-N-acetylmuramoyl-pentapeptide-transferase, which are more closely related to cell wall synthesis. These changes support the hypothesis that Groups 1 and 2 require more use of pathways associated with repairing immune damage, whereas Group 3 is more focused on growth.

Subsequently, we compared metabolic flux differences between Groups (Fig 6). We found higher CTP synthesis in Group 2 *vs* Group 3, but lower Cysteine synthesis and Cytidylate kinase. We also see the changes in Phospho-N-acetylmuramoyl-pentapeptide-transferase noted previously. Once again, this supports the conclusion that Groups 2 and 3 are more similar to each other than to Group 1. While both Groups show upregulation to pathways associated with growth and repair, Group 3 shows higher relative upregulations than the fluxes in Fig 4 in growth pathways.

### Determining the protein expression profiles of *S*. *enterica* during infection

**Fig 7:**
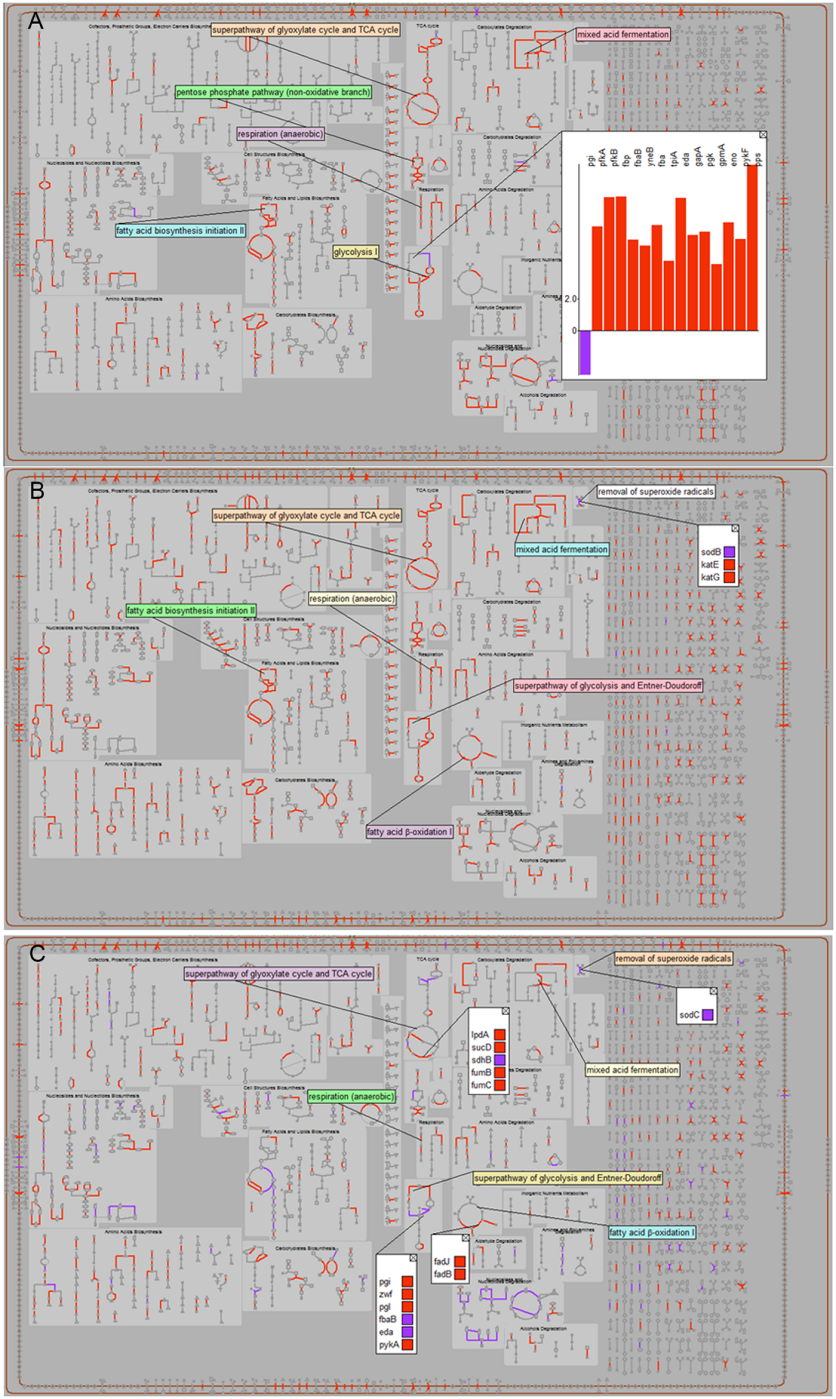
Visualization of the proteome indicating up and down regulated pathways. Up-regulated pathways are highlighted in red and down-regulated pathways are highlighted in blue. (A) The perturbed pathways in Group 2 *S*. Typhimurium relative to Group 1 *S*. Typhimurium. (B) The perturbed pathways in Group 3 *S*. Typhimurium relative to Group 1 *S*. Typhimurium. (C) The perturbed pathways in Group 3 *S*. Typhimurium relative to Group 2 *S*. Typhimurium.

We complemented the RNA-Seq gene expression work and the metabolic modelling by examining the proteome expressed by *Salmonella* in each host environment. We developed an immunomagnetic isolation method for the purification of *S*. Typhimurium from the organs of infected mice. We looked at the relative protein expression levels of *S*. Typhimurium in Group 1, Group 2 and Group 3 compared to the *in vitro* input (S12 Table) and also compared the DE proteins (log_2_ fold change >2.0) between the different groups. The numbers of the DE proteins are shown in S13 Table. Fig 7 shows the perturbed pathways in *S*. Typhimurium in the different Groups. S13 Table indicates that for Group 1 *vs* Group 2 *Salmonella* there were 68 up-regulated and 450 down-regulated proteins, for Group 1 *vs* Group 3 *Salmonella* there were 37 up-regulated and 1,020 down-regulated proteins, and for Group 2 *vs* Group 3 there were 147 up-regulated and 683 down-regulated proteins.

The relative protein expression changes in Group 1 *Salmonella* compared to Group 2 *Salmonella* indicate up-regulation of proteins with roles in virulence (ClpP, EcnB, RcsF, SseA, SspA, TatA), stress (SodB) and antimicrobial peptide resistance (PagP). Conversely, the up-regulated proteins in Group 2 *Salmonella* compared to Group 1 *Salmonella* have roles in aminoacyl tRNA biosynthesis, RNA degradation, two component systems and some key metabolic pathways (S12 and S13 Tables, Fig 7A). Broadly, these results show concordance with the findings from our transcriptomic data.

Our proteomics data also show that proteins with roles in virulence (Lrp, SseA, SspA), stress (DksA, SodB), iron (Ftn) and antimicrobial peptide resistance (PagP) were up-regulated in Group 1 *Salmonella* compared with Group 3. The up-regulated proteins in Group 3 relative to Group 1 include those involved in translation, cellular amino acid biosynthetic process, pathogenesis (Hfq, InvB, InvH, InvG, LppB, OmpA, OmpC, OmpF, PhoQ, PipC, PrgK, SipA, SipB, SipC, SopB, StpA, TSX), entry into host cell, phosphotransferase system (Crr, FruA, FruB, ManY, ManZ, MtlA, NagE, PtsG, PtsH, PtsI, PtsN, PtsP, SgaB) and other biological processes (S12 and S13 Tables, Fig 7B). Many key metabolic processes were up-regulated in Group 3 *Salmonella* compared to Group 1 *Salmonella*, including, RNA degradation (DeaD, Hfq, PcnB, Ppk, RhlB), bacterial secretion system (Ffh, FtsY, IinG, PrgK, SecD, SecF, SecG, SsaN TatA, YajC, YidC), chemotaxis (CheA, CheM, CheW, DppA, MalE, MglB, Tcp, Tsr), peptidoglycan biosynthesis (DacC, Ddl, FtsI, MurA, MurD, MurF, MurG, MrdA), biosynthesis of siderophores (EntB, EntC, EntE, EntF), and phosphotransferase system (FruA, FruB, ManZ, NagE, PtsA, PtsG, PtsH, PtsN, PtsP) (S12 and S13 Tables, Fig 7C). The finding that protein biosynthesis was up-regulated in Group 3 bacteria compared to Group 1 bacteria in the proteomics data is in agreement with the transcriptomic data.

We also found concordance between the Group 2 *vs* Group 3 proteomic and transcriptomic data. Protection against oxidative stress was important in *Salmonella* from Group 2, while adaptation to nutritional carbon and iron stress was dominant in *Salmonella* from Group 3. The data indicates that proteins with roles in amino sugar and nucleotide sugar metabolism, antioxidant activity (AhpX, DksA, SodC, Tpx, TsaA), ferric iron binding (Bfr, Dps, Ftn), virulence (BasR, OmpR, PagC, PhoP) and resistance to reactive oxygen species (ArcA) were significantly up-regulated in Group 2 *Salmonella* compared to Group 3 *Salmonella*.

### Determining the gene expression profiles of the host during infection with *S*. Typhimurium

**Fig 8:**
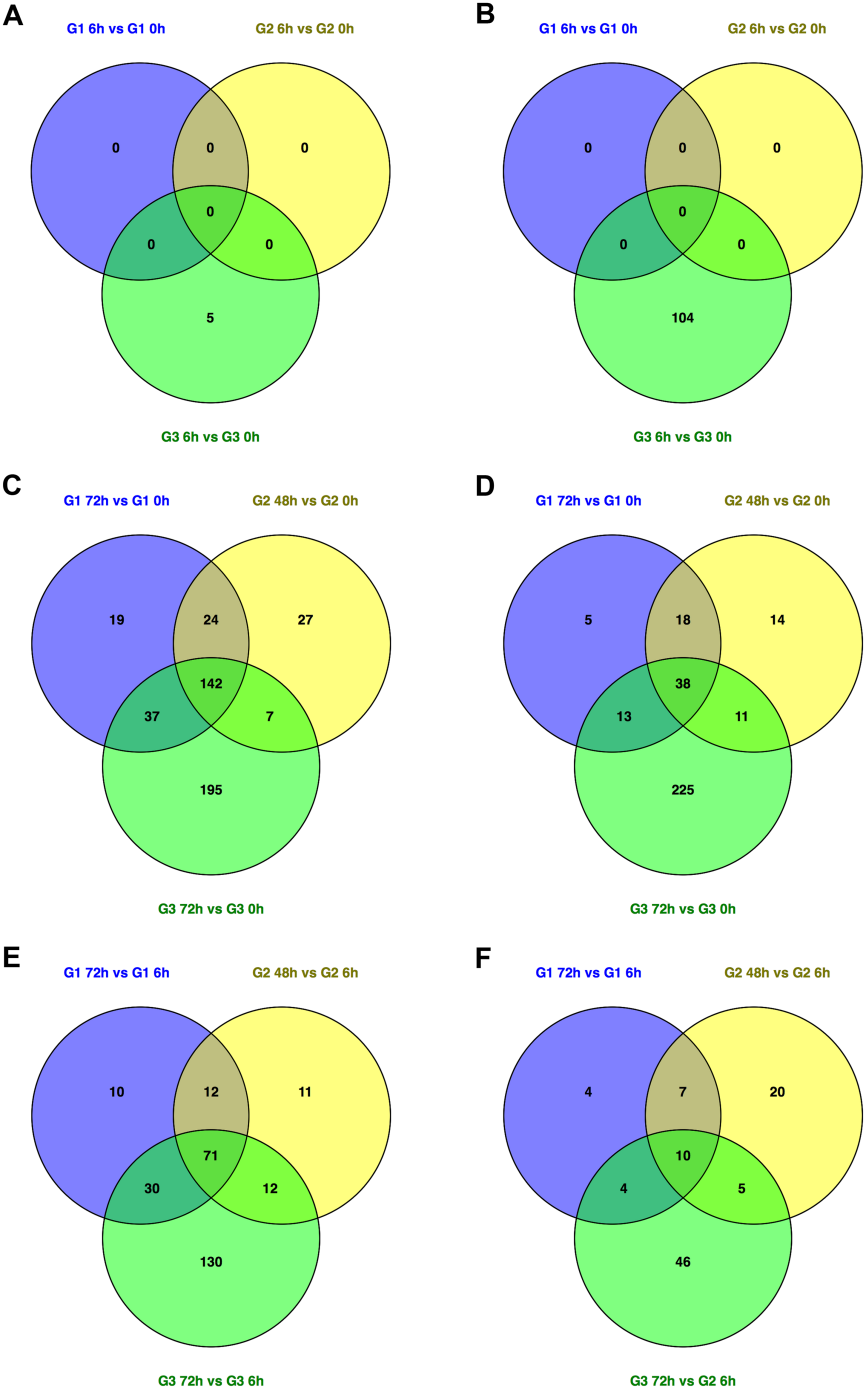
Visualization of the DE host genes. Venn diagrams show (A) Up-regulated DE genes for each Group at the 6 h *vs* 0 h comparison. (B) Down-regulated DE genes for each Group at the 6 h *vs* 0 h comparison. (C) Up-regulated DE genes for each Group at the end point of the infection (72 h for Group 1 and Group 3, and 48 h for Group 2) *vs* 0 h comparison. (D) Down-regulated DE genes for each Group at the end point of the infection (72 h for Group 1 and Group 3, and 48 h for Group 2) *vs* 0 h comparison. (E) Up-regulated DE genes for each Group at the end point (72 h for Group 1 and Group 3, and 48 h for Group 2) *vs* 6 h comparison. (F) Down-regulated DE genes for each Group at the end point of the infection (72 h for Group 1 and Group 3, and 48 h for Group 2) *vs* 6h comparison.

**Fig 9:**
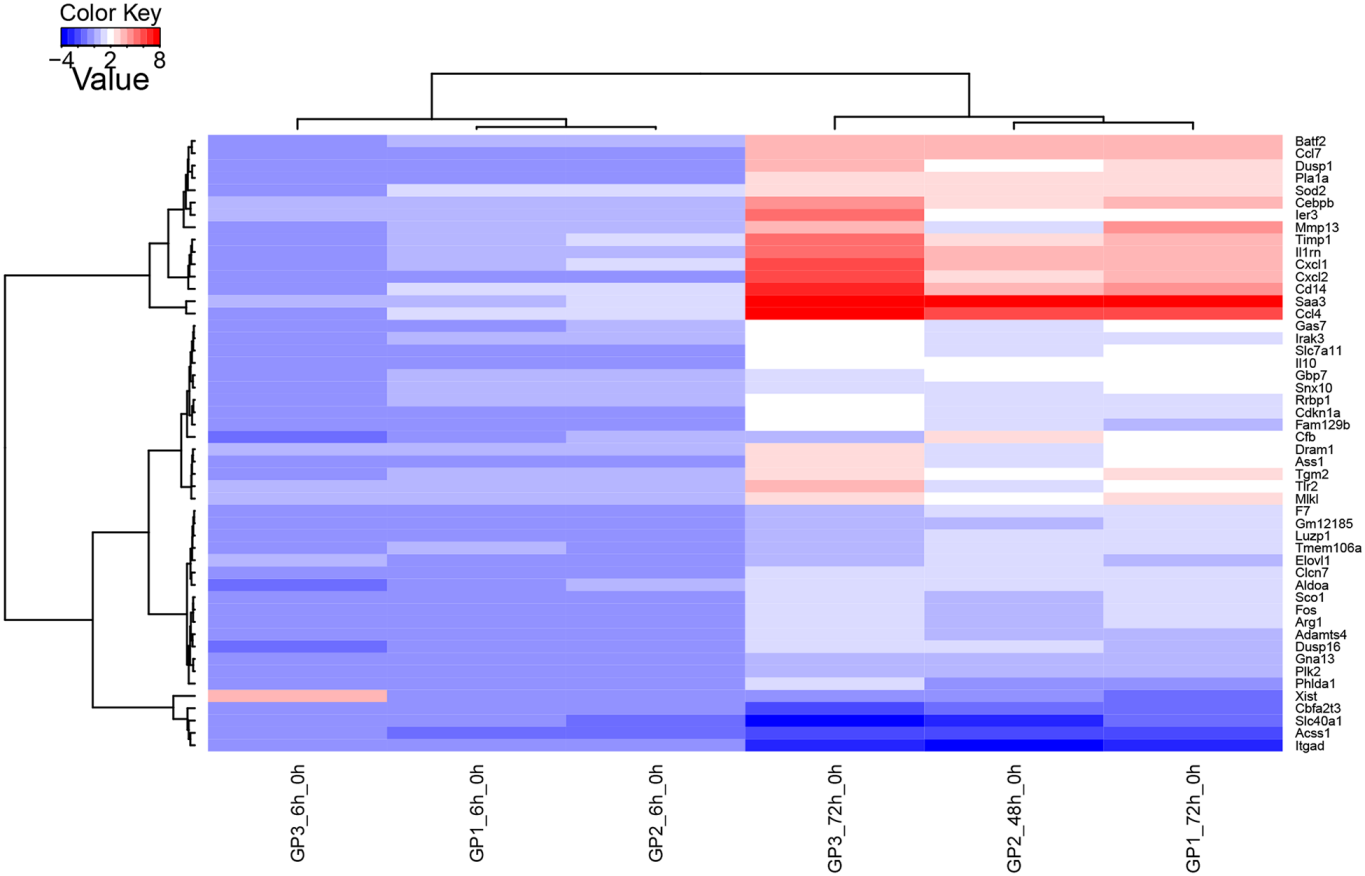
Heatmap of the top 50 most DE genes in Group 1, Group 2 and Group 3 mice at 6h and 48 or 72h p.i., compared to the uninfected mice. The dendrograms are based on Ward’s method using scaling between sample profiles. Red and blue colours represent up- and down-regulation, respectively. Genes are visualized in rows, and Groups are in the columns. Patterns of up- and down-regulated genes are depicted in separate clusters.

As well as studying the pathogen, we were also interested in the host response to the infection, and we used microarrays to study the gene expression profiles from the mice during each of the infections (S14 Table). The microarray datasets of the different murine hosts/experimental conditions were analysed to determine the presence of distinct and overlapping clusters of gene expression and to gain insight into the host response to infection (Figs 8 and 9, S15–S19 Tables). The number of DE genes and the functional categories of these genes are given in S15 Table. There were a similar number of DE genes in Group 1 and Group 2, but substantially more DE genes in Group 3.

### Comparing DE host genes between Groups

The heatmap of the top 50 most DE genes in Group 1, Group 2 and Group 3 at 6h and 48h or 72h, compared to the uninfected mice (Fig 9), highlights the strongest transcriptional profiles occurring in the three groups, mainly at 48 or 72 h p.i. and to a lesser extent 6 h p.i. The profiles reveal some important genes in separate clusters, among which are the strongly up-regulated genes (encoding Batf2, Ccl7, Pla1a, Sod2, Cebpb, Timp1, II1m, Cxcl1, Cxcl2, Cd14, Saa3 and Ccl4) and down-regulated genes (encoding Itgad, Acss1, Slc40a1, Cbfa213 and Xist) that are consistent across Group 1, Group 2 and Group 3 mice (Fig 9) at 48 or 72 h p.i. The known biological contexts indicated by these immunity-related genes include acute-phase response (SAA3), regulation of apoptosis (CebpB, Sod2, Timp1), cellular response to stress (Sod2), chemotaxis (Ccl4 and Ccl7), regulation of transcription (Batf2, Cebpb), purine ribonucleotide binding (Acss1) and membrane process (Slc40a1).

Further comparative analyses revealed differences among the different Groups. Fig 8 depicts the comparisons of groups at 6h *vs* 0h (Fig 8A and 8B), 48h *vs* 0h or 72h *vs* 0h (Fig 8C and 8D) and 48h *vs* 6h or 72h *vs* 6h (Fig 8E and 8F). For Group 1 and Group 2 there were no DE genes when comparing between the 6 h and 0 h time points. This might indicate that in the early phase of the infection, the host is able to mount a sufficient response with the innate immune system. However, it is also possible that there are DE genes, but that the levels of expression are not statistically significant. For the Group 3 comparison between 6 h compared to 0h, there were 109 DE genes, of which 5 were up-regulated and 104 were down-regulated (S15 Table). There were no common pathways for the up-regulated genes, however, “Antigen processing and presentation” was significantly overrepresented in the pathway analyses for the down-regulated genes in Group 3 (6 h *vs* 0 h) (S15 Table).

There were many more DE genes for each of the Groups when comparing the end time point (72 h for Group 1 and Group 3, and 48 h for Group 2) to the uninfected mice (296 DE genes in Group 1; 281 DE genes in Group 2; and 668 DE genes in Group 3; S15 Table). Pathway analysis showed a significant over-representation of “Cytokine activity”, “Chemokine signaling pathway”, “Toll-like receptor signaling pathway” and “NOD-like receptor signaling pathway” in the up-regulated genes in each grouping (S15 Table). There were no common pathways for the down-regulated DE genes between the comparisons (S15 Table).

Compared to the end time point (72 h for Group 1 and Group 3, and 48 h for Group 2) *vs* uninfected (0 h), there were fewer DE genes for each of the Groups when comparing the end time point to the 6 h time point (148 DE genes in Group 1; 148 DE genes in Group 2; and 308 DE genes in Group 3; S15 Table). The statistically enriched pathways for the up-regulated genes in each group are “Chemokine signaling pathway”, and “NOD-like receptor signaling pathway” (S15 Table). There were no common pathways for the down-regulated DE genes between the group comparisons.

Between 6 h and 72 h p.i., Group 3 mice compared to Group 1 mice (6 h and 72 h p.i.) and Group 2 mice (6 h and 48 h p.i.), showed significant induction of Toll-like receptor signaling pathways (S16 Table). In contradistinction, the Group 2 mice data demonstrated unique downregulation of genes involved in NOD-like receptor signalling pathway, chemokine signalling pathway and Toll-like receptor signalling pathway between 6 h and 48 h post infection (S16 Table). Genes encoding proteins with GTP or GTPase activities (RAB32, GVIN1, GBP10, IFI47, TUBB6 and TGTP1) and other immunity-related genes (CFB, LILRB4 and CCL9,) are commonly upregulated in Group 1 mice (0 hr to 72 post infection) and Group 2 mice (0 hr to 48 post infection). The upregulated LILRB4 gene may play a role in limiting the inflammatory response during infection [23]. However, there is an overlap in the significantly depressed specific GTPase activator activity (HMHA1, ARHGEF1 and RASA3; that play roles in the regulation of Rho GTPases) in both Group 1 and Group 3 between 0 h and 72 h post infection.

## Conclusion

In this study, we have used different infection conditions to explore the host-pathogen responses during infection. The global transcriptome data presented in this study, reflects the ‘average’ gene expression profile occurring in the entire population of *Salmonella* and host cells, and this may not represent accurately the complex heterogeneity of individual host and pathogen cells during infection. For example, a recent study by Saliba *et al*. performed single-cell RNA-seq of *Salmonella* infected *in vitro* grown macrophages and demonstrated gene expression heterogeneity among infected macrophages related to the growth rate of the bacteria [24]. A challenge for the future will be to determine the gene expression profiles of individual bacteria and host cells from *in vivo* samples. However, while single cell RNAseq is a high-resolution technique that can be applied to study what is happening in a single cell to environmental signals; It may be not sufficient to study what is happening during an infection because of the requirement to perform single cell sequencing of a very large number of cells for the different cellular heterogeneities and ultra/microenvironments that the bacteria will encounter in the host. Therefore, we believe that the classic multi-cell analysis, together with clear disadvantages, has the advantage of providing a measure of the variance, *i*.*e*. all the transcriptional programs the bacteria. Ultimately, combination of single-cell, sub-population and whole-organ analyses will be required to fully determine the host-pathogen behaviour during infection. This might provide knowledge and technological basis for targeting individual bacterial components *in vivo* with novel drugs and vaccines and for eliciting immune responses against individual bacterial virulence determinants directed at the sites of infection where these are maximally expressed by the bacteria.

## Materials and methods

### Ethics statement

All animals were handled in strict accordance with good animal practice as defined by the relevant international (Directive of the European Parliament and of the Council on the protection of animals used for scientific purposes, Brussels 543/5) and local (Department of Veterinary Medicine, University of Cambridge) animal welfare guidelines. All animal work was approved by the ethical review committee of the University of Cambridge and was licensed by the UK Government Home Office under the Animals (Scientific Procedures) Act 1986, Project Licence 80/2572. (S1 File)

### Bacterial strains and growth conditions

*S*. Typhimurium strain SL1344, is a virulent wild-type strain that has an LD_50_ by the i.v. route of <20 colony forming units (CFU) for innately susceptible mice [25]. Bacterial cultures for infection were grown from single colonies in 10 ml Luria-Bertani (LB) broth incubated overnight without shaking at 37°C, then diluted in phosphate buffered saline (PBS) to the appropriate concentration for inoculation.

### Murine infections

Mice were maintained in the University facility with a 12 hour light/12 hour dark cycle at 21±2°C, with lights on at 08:00h (GMT), they were housed in individually ventilated cages, were provided with Aspen chip bedding with environmental enrichment and nesting, and they received water and food *ad libitum*. Sex- and aged-matched 9–12 week old C57BL/6 wild type mice (Harlan Olac Ltd), and *gp91*^*-/-*^*phox* mice (bred at the Wellcome Trust Sanger Institute, Hinxton, Cambridge, United Kingdom) were randomly divided into groups. Mice were infected by i.v. injection of bacterial suspensions in a volume of 0.2 ml. Inocula were enumerated by plating dilutions onto LB agar plates. Animals that received the same infection were housed together with up to 10 mice per cage. Mice were monitored twice daily, and culled by cervical dislocation at the pre-determined time points. Upon culling, the animals livers and spleens were aseptically removed and processed as described here and below. In order to calculate the number of viable bacteria in the sample, the organ was homogenized in sterile water using a Colworth Stomacher 80. The resulting homogenate was diluted in a 10-fold series in PBS and LB agar pour plates were used to enumerate viable bacteria. All of the animals were infected between 09:00 and 12:00 GMT. All of the animals were culled between 09:00 and 12:00 GMT. The number of mice per experimental group and for each experiment is defined below, and summarised in S19 Table and S1 File.

### Generation and transfer of *in vivo* grown SL1344

To generate the *in vivo* grown SL1344, three C57BL/6 mice were infected i.v. with log_10_ 4.0 CFU of *S*. Typhimurium SL1344 and killed 72 h p.i., when the bacterial load in the spleens was Log_10_ 7.19 ± 0.22 CFU (error given as standard deviation). The three infected spleens were homogenized together, using an Ultra-Turrax T25 blender in 30 ml of distilled water. 9.4 ml of organ homogenate was added to 30.6 ml of PBS, which was further diluted by 10-fold serial dilutions in PBS prior to i.v. inoculation. The transfer of bacteria to the first naïve animal of the group was completed in less than 2 min from the death of the donor mice.

### Prokaryotic RNA preparation for RNA-seq

*In vitro* grown SL1344 cultures were fixed with 2 volumes of RNA protect Bacteria (Qiagen) and harvested by centrifugation for 4 min at 13,000 rpm. For the *in vivo* grown SL1344, 30 mice were killed by cervical dislocation and the spleens were removed. Six batches of five spleens were homogenised in 8 ml of distilled water). Two 8ml organ homogenates (i.e. organ homogenates from 10 spleens) were combined, this was repeated twice more to cover the six batches of five spleens, and incubated with 2 volumes of RNA Protect (Qiagen). Bacteria were harvested by passing the suspension through a 40 μM filter followed by centrifugation for 4 min at 13,000 rpm. RNA was isolated from each pellet (*in vitro* grown or *in vivo* grown bacteria) using the SV RNA isolation kit (Promega) according to the manufacturer’s instructions. RNA was prepared for ~Log_10_ 10.32 CFU of bacteria used as the input for Group 1; ~Log_10_ 8.67 CFU of Group 1 bacteria; ~Log_10_ 9.15 CFU of Group 2 bacteria; ~Log_10_ 10.52 CFU of bacteria used as the input for Group 3; and ~Log_10_ 9.81 CFU of Group 3 bacteria. 23S and 16S rRNAs were depleted using a MicrobExpress kit (Ambion). Genomic DNA was removed with two digestions using Amplification grade DNAse I (Invitrogen), to below PCR-detectable levels. RNA was reverse transcribed using random primers (Invitrogen) and Superscript III (Invitrogen) at 42°C for 2 h and denatured at 70°C for 20 min. Resulting cDNA was cleaned using an Illustra Autoseq G-50 column (GE Healthcare). *rpoB* (cc444 5’-cctgagcaaagacgacatca-3’ and cc445 5’-tggcgttgatcatatcctga-3’), *recA* (cc446 5’-tttcactggacatcgcactc-3’ and cc447 5’-gtatccggctgagagcagag-3’) and *proS* (cc448 5’-ctctggtcgatacgccaaat-3’ and cc449 5’-taattacggcgcgaatctct-3’) were used as targets for a PCR as a positive control for reverse transcription. cDNA was subjected to Illumina sequencing.

### Sequencing, read mapping and RNA-Seq analysis

Library construction and sequencing were carried out as previously described [26]. Illumina sequence reads were mapped to the *S*. Typhimurium SL1344 genome sequence (GenBank accession: FQ312003) using the Burrows Wheeler Alignment protocol [27]. Mapped reads obtained are summarized in Table 1. Identification of DE genes was carried out using DESeq [28] bioconductor package in R statistical package environment (version 3.0.2). In order to increase the statistical detection power of the RNA-Seq datasets we applied DESeq independent filtering to the raw mapped read counts of genes in all samples. The filtering step removed the genes in the lowest 40% quantile of overall sum of read counts. The filtered read counts were then normalized to reduce variation between samples by transforming the data to a common scale using computed size factors. After computing the dispersions in the replicate samples, we used the negative binomial distribution model to obtain P-values for identifying differentially expressed genes. Differentially expressed genes were selected based on log_2_-fold change ≤ 1.5 or ≥ 1.5 and FDR = 10%.

### Analysis of differential expression

Identification of differentially expressed genes was carried out using DESeq [28] bioconductor package in R statistical package environment (version 3.0.2). In order to increase the statistical detection power of the RNA-Seq datasets we applied DESeq independent filtering to the raw mapped read counts of genes in all samples. The filtering step removed the genes in the lowest 40% quantile of overall sum of read counts. The filtered read counts were then normalized to reduce variation between samples by transforming the data to a common scale using computed size factors. After computing the dispersions in the replicate samples, we used the negative binomial distribution model to obtain P-values for identifying differentially expressed genes. Differentially expressed genes were selected based on log_2_-fold change ≥ 1.5 and FDR = 10%.

### Eukaryotic RNA preparation

Post mortem spleens were dissected into small pieces that were transferred to, and stored in, RNAlater solution (Qiagen). RNA was extracted and purified using RNeasy MinElute spin columns (Qiagen), as per the manufacturers’ instructions.

### Microarray analysis

RNA from C57BL/6 (Group 1), C57BL/6 (Group 2) and *gp91phox*^-/-^ (Group 3) mice were used for microarray analysis. After quality checks, RNA samples were hybridized onto Illumina MouseWG-6 v2.0 Expression BeadChip (version 3.1). After hybridization, chips were scanned on an Illumina BeadArray Reader and raw intensities were extracted using Illumina BeadStudio Gene Expression Module. Microarray analysis was carried out in R statistical package environment (www.r-project.org). The background corrected probe intensities imported from BeadStudio into R, using lumiR, were transformed to log2 and normalized using quantile normalization using lumiT and lumiN functions, respectively. The tests for statistical difference between two conditions were performed using the empirical Bayes method (eBayes) implemented in limma [29]. Probability values were adjusted using the Benjamini-Hochberg method [30] for multiple hypothesis correction. Differential gene expression was referred to as significant, if the adjusted P-value was < 0.05 and the log_2_ fold change was ≥ 1.5 or ≤ 1.5.

### Protein samples and proteomics

*In vitro* grown bacteria were harvested from 48 ml of culture (bacteria were grown as previously described for RNA preparation for RNA-seq) by centrifugation in a micro-centrifuge at 13,000 rpm for 2 min. The supernatant was removed by aspiration, and the pellets were resuspended in SDW containing 170μg/ml of chloramphenicol (to inhibit protein synthesis). The washing and centrifugation was repeated three times.

For the *in vivo* grown SL1344, 12 mice were killed by cervical dislocation and the spleens were removed and homogenised in 30 ml of distilled water containing 170μg/ml of chloramphenicol. The homogenate was passed through a 40mM filter followed by centrifugation for 2 min at 13,000 rpm. The supernatant was removed by aspiration, and the pellets were resuspended in SDW containing 170μg/ml of chloramphenicol. To remove host cell debris, the samples were centrifuged at 1,800 rpm for 2 min the supernatant was removed and transferred to a fresh tube. Bacteria were harvested by centrifugation in a micro-centrifuge at 13,000 rpm for 2 minutes. The supernatant was removed by aspiration, and the pellets were resuspended in SDW containing 170μg/ml of chloramphenicol. The washing and centrifugation was repeated three times.

Subsequently, for the *in vitro* and *in vivo* grown SL1344, the bacteria were harvested by centrifugation in a micro-centrifuge at 13,000 rpm and 4°C for 2 minutes. The supernatant was removed by aspiration, and the pellets were resuspended with anti-*Salmonella* Dynabeads (Invitrogen) containing 170μg/ml of chloramphenicol and incubated for 20 min at room temperature with constant mixing. Dynabead-bacteria were collected by placing the tube next to a magnet for 2 min and removing the supernatant, and the pellets were resuspended to homogeneity in wash buffer containing 170μg/ml of chloramphenicol. The collection and wash step was repeated twice more; on the final resuspension, the Dynabead-bacteria were resuspended in 20μl urea buffer.

Protein samples were resuspended in 2 x Final Sample Buffer (FSB) (0.125 M Tris-HCl (pH 6.8), 4% SDS, 20% glycerol, 10% 2-Mercaptoethanol, 0.05% bromophenol blue), reduced with DTT and alkylated with iodoacetamide prior to separation in a 4–12% NuPAGE Bis-Tris gel (Invitrogen). Gels were stained with colloidal Coomassie blue (Sigma).

Each gel lane was excised to 12 bands followed by in-gel digestion with trypsin overnight at 37°C. Peptides were extracted with 0.5% formic acid (FA)/50% CH_3_CN and dried in a SpeedVac (Thermo Fisher). The peptides were re-suspended in 0.5%FA/100% H_2_O just before the LC-MS/MS analysis on an Ultimate 3000 RSLCnano System coupled to an LTQ Orbitrap Velos hybrid mass spectrometer equipped with a nanospray source. The peptides from each band were first loaded and desalted to a PepMap C18 nano-trap (100 μm i.d. x 20 mm, 100Å, 5μm) at 10 μL/min for 15 min, then separated on a PepMap RSLC C18 column (75 μm i.d. x 500 mm, 100 Å, 2 μm) at a linear gradient of 4–32% CH_3_CN/0.1% FA) in 90min with a total cycle time of 120 min at a flow rate at 300 nL/min. The HPLC, columns and mass spectrometer were all from Thermo Fisher Scientific. The Orbitrap mass spectrometer was operated in the standard “top 10” data-dependent acquisition mode while the preview mode was disabled. The MS full scan was set at m/z 380–1,800 with the resolution at 60,000 at m/z 400 and AGC at 1x10^6^ with a maximum injection time at 200 msec. The siloxane ion at 445.120030 was used as lock mass. The 10 most abundant multiply-charged precursor ions (z ≥ 2), with a minimal signal above 1,000 counts, were dynamically selected for CID (Collision Induced Dissociation) fragmentation in the ion trap, which had the AGC set at 7,000 with the maximum injection time at 300 msec. The precursor isolation width was set at 2 Da. The normalized collision energy for CID MS/MS was set at 35%. The dynamic exclusion duration time for selected ions for MS/MS was set for 60 sec with ±0.5 Da exclusion mass width.

### MS/MS data processing, protein identification and pathway analysis

The raw files were processed in Proteome Discoverer (Version 1.3, Thermo Fisher) with Mascot (Version 2.3, Matrix Science) as the search engine. The *S*. Typhimurium SL1344 protein database was extracted from SL1344 embl file (version 2011) and the mouse protein database is an IPI database (version May 2012). The Mascot search used following parameters: trypsin with maximum 3 missed cleavages sites; peptide mass tolerance 10 ppm, MS/MS fragment mass tolerance at 0.49 Da, and variable modifications of Acetyl (Protein N-term), Carbamidomethyl (C), Deamidated (NQ), Oxidation (M), Dioxidation (M), Oxidation (W), Gln->pyro-Glu (N-term Q) and Methyl (E). Peptides were filtered by the PEP (Posterior Error Probabilities) score at 0.0100 by the Percolator in Mascot, and significance threshold at 0.05. The protein content calculation (weight %) used emPAI score and protein molecular weight [31]; when multiple protein IDs matched the same set of peptides, only one entry was used. These parameters were used to determine relative protein expression in different experimental groups according to their relative abundance. DAVID pathway clustering [32] was used to identify statistically enriched GO terms and KEGG pathways in the sets of DE proteins using 5% FDR as a cut-off.

### Gene expression constrained metabolic modelling

Our procedure to create and evaluate a gene-expression constrained metabolic model can be divided into three parts: preparing the raw RNA values for metabolic modelling *via* transformation and normalization, combining and integrating gene expressions with the metabolic model by first working out per-reaction expressions, and then constraining these reactions by these values, and finally finding fluxes in the resulting metabolic model, *via* flux balance analysis. In addition, we used an automated procedure for processing and filtering of results, since there were too many for manual examination. Full details can be found online at https://github.com/maxconway/salmonella-mouse.

### Preparing RNA values for metabolic modelling

The sequence count data set consists of 11 measurements for each of 6,579 genes: three technical replicates for each of the three *in vivo* conditions, and two technical replicates for the *in vitro* input condition. The logarithm of the sequence count plus one was generated. The logarithm was used because the sequence counts are approximately log-normally distributed and one is added because some of the counts are zero, particularly in the *in vitro* experiments where the values are generally much smaller. The replicates were normalised against their mean, to remove technical and experimental differences, followed by identification of the mean of the two or three replicates for each gene within each experimental group. Subsequently, each group was normalised against the *in vitro* input as a control. Finally, the standard deviation was adjusted to 0.1. The resulting values were approximately normally distributed around one, but with a spike at one.

### Combining and integrating gene expressions for the metabolic model

We use a metabolic network with Gene-Protein-Reaction mappings from the literature. To combine this with the RNA data, we mapped each gene in the RNA dataset to the equivalent gene in the metabolic model. Where multiple genes correspond to a particular reaction, we combined them *via* a continuous extrapolation of the boolean function encoded in the Gene-Protein-Reaction mappings. Specifically, if *both* of the genes or gene sets are required (*i*.*e*. an `AND`relation), we took the minimum activation of the two; if *one* of the genes or gene sets are required (*i*.*e*. an `OR`relation), we took the maximum activation of the two. Where there was no information about a gene, we assumed that it is abundant by default, leaving the other gene in the expression to dictate the output level.

### Finding fluxes in the metabolic model based on gene expression

Standard flux balance analysis was used to find the base flux levels without any gene expression restrictions. Next, we calculated a new flux "target" for each group by multiplying the base flux level by the activation, and set new flux bounds as 10% of this target on either side. Based on these new flux bounds, we conduct our final round of flux balance analysis to find the actual predicted fluxes.

### Processing and filtering results for the metabolic model

This procedure gives around 10,000 predicted fluxes (2,500 reactions in the model, and four groups). This was too many to examine manually, so we used a filtering procedure to find the most important. The filtering procedure first removed reactions with unacceptably low stability, that is when reactions were sensitive to initial conditions. This is important because the size of the model means that some combinations of reactions are undetermined, so that there are multiple valid solutions with different flux values. This filtering was conducted by a monte-carlo approach, with reactions removed where they displayed a raw intra-group standard deviation of greater than 10^−5^, or if they displayed a coefficient of variation of greater than 10% for the control group.

Next, reactions were filtered to remove those where the experimental groups were all near the control group, and finally, reactions were ranked by their standard deviation, in order to find those that had the most “interesting” results.

### Accession numbers

The complete RNA-Seq dataset from this study has been deposited in the ArrayExpress database (www.ebi.ac.uk/arrayexpress) with the accession number E-ERAD-78. The microarray data can be accessed as through ArrayExpress at E-MTAB-5494. The complete proteomic dataset from this study has been deposited in the PRIDE database (www.ebi.ac.uk/pride/q.do) with the accession number PXD005855.

## Supporting information

S1 Table Complete RNA-Seq transcriptome dataset for all bacterial genes and intergenic regions. (XLS)

S2 Table The number of DE bacterial genes for each experimental condition. The pathways that were statistically overrepresented among the DE genes are listed for each of the datasets compared.
(XLS)

S3 Table Comparing the RNA-Seq transcriptome of *S*. Typhimurium SL1344 from Group 1 *vs* Input. Table showing the DE genes from the Group 1 *S*. Typhimurium *vs in vitro* Input comparison.
(XLS)

S4 Table Comparing the RNA-Seq transcriptome of *S*. Typhimurium SL1344 from Group 2 *vs* Input. Table showing the DE genes from the Group 2 *S*. Typhimurium *vs in vitro* Input comparison.
(XLS)

S5 Table Comparing the RNA-Seq transcriptome of *S*. Typhimurium SL1344 from Group 3 *vs* Input. Table showing the DE genes from the Group 3 *S*. Typhimurium *vs in vitro* Input comparison.
(XLS)

S6 Table Unique and shared DE bacterial genes for each Group *vs in vitro* Input and Group *vs* Group comparison. Table showing the unique and shared DE genes from each Group *vs in vitro* Input, and Group *vs* Group comparison.
(XLS)

S7 Table The number of DE bacterial genes between the experimental conditions. The pathways that were statistically overrepresented among the DE genes are listed for each of the datasets compared.
(XLS)

S8 Table Comparing the RNA-Seq transcriptome of *S*. Typhimurium SL1344 from Group 1 *vs* Group 2. Table showing the DE genes from the Group 1 *S*. Typhimurium *vs* Group 2 *S*. Typhimurium comparison.
(XLS)

S9 Table Comparing the RNA-Seq transcriptome of *S*. Typhimurium SL1344 from Group 1 *vs* Group 3. Table showing the DE genes from the Group 1 *S*. Typhimurium *vs* Group 3 *S*. Typhimurium comparison.
(XLS)

S10 Table Comparing the RNA-Seq transcriptome of *S*. Typhimurium SL1344 from Group 2 *vs* Group 3. Table showing the DE genes from the Group 2 *S*. Typhimurium *vs* Group 3 *S*. Typhimurium comparison.
(XLS)

S11 Table Abbreviations and definitions for legends in Figs 4, 5 and 6. (XLS)

S12 Table Proteome datasets, and up- and down-regulated protein expression between groups. Table showing the lists of proteins, protein content (weight %) and the relative protein expression (log_2_ fold-change) of *S*. Typhimurium in Group 1, Group 2, and Group 3 compared to the *in vitro* grown Input.
(XLS)

S13 Table The number of differentially expressed bacterial proteins for each experimental condition. The pathways that were statistically over-represented among the DE proteins are listed for each of the datasets compared.
(XLS)

S14 Table Microarray transcriptome dataset and relative gene expression levels for host genes and intergenic regions. Table showing the relative gene expression (log_2_ fold-change) in Group 1, Group 2, and Group 3 mice compared to the *in vitro* grown Input and host Group *vs* Group comparisons, at different time points.
(XLS)

S15 Table The number of DE host genes for each experimental condition. The pathways that were statistically overrepresented among the DE genes are listed for each of the datasets compared.
(XLS)

S16 Table The number of DE host genes between the experimental conditions. The pathways that were statistically overrepresented among the DE genes are listed for each of the datasets compared.
(XLS)

S17 Table Summary of the up- and down-regulated DE host genes. Table showing *In vivo vs In vivo* comparisons.
(XLS)

S18 Table Unique and shared DE host genes for each Group *vs* Group comparison. Table showing the unique and shared DE genes from each Group *vs* Group comparison.
(XLS)

S19 Table Study design indicating the number of animals per group/experiment. (XLSX)

S1 File NC3Rs ARRIVE guidelines checklist. (PDF)

## Abbreviations

CFU: colony forming units
DE: differentially expressed
FDR: false discovery rate
FSB: final sample buffer
GO: gene ontology
i.v.: intravenously
LB: Luria-Bertani
LD: lethal dose
MS: mass spectrometry
NTS: non-typhoidal Salmonella
PBS: phosphate buffered saline
p.i.: post infection
ROS: reactive oxygen species
SDW: sterile distilled water
SPI: *Salmonella* pathogenicity island
